# Peer influence in adolescent drinking behavior: A meta-analysis of stochastic actor-based modeling studies

**DOI:** 10.1371/journal.pone.0250169

**Published:** 2021-04-16

**Authors:** Valeria Ivaniushina, Vera Titkova

**Affiliations:** Department of Sociology, National Research University Higher School of Economics, Saint Petersburg, Russian Federation; Geneva University Hospitals, SWITZERLAND

## Abstract

**Objectives:**

To measure the effects of peer influence and peer selection on drinking behavior in adolescence through a rigorous statistical approach designed to unravel these interrelated processes.

**Methods:**

We conducted systematic searches of electronic databases, thesis collections and conference proceedings to identify studies that used longitudinal network design and stochastic actor-oriented modeling to analyze drinking behavior in adolescents. Parameter estimates collected from individual studies were analyzed using multilevel random-effects models.

**Results:**

We identified 26 articles eligible for meta-analysis. Meta-analyses for different specifications of the peer influence effect were conducted separately. The peer influence effect was positive for every specification: for average similarity (avSim) mean log odds ratio was 1.27 with 95% confidence interval [0.04; 2.49]; for total similarity (totSim) 0.46 (95% CI = [0.44; 0.48]), and for average alter (avAlt) 0.70 (95% CI = [-0.01; 1.41]). The peer selection effect (simX) was also positive: 0.46 (95% CI = [0.28; 0.63]). Conversion log odds ratio values to Cohen’s d gives estimates from 0.25 to 0.70, which is considered as medium to large effect.

**Conclusions:**

Advances in methodology for social network analysis have made it possible to accurately estimate peer influence effects free from peer selection effects. More research is necessary to clarify the roles of age, gender, and individual susceptibility on the changing behavior of adolescents under the influence of their peers. Understanding the effects of peer influence should inform practitioners and policy makers to design and deliver more effective prevention programs.

## Introduction

Alcohol consumption at a young age is a global public health problem that leads to multiple immediate and long-term detrimental consequences [1–4]. Understanding the factors associated with alcohol consumption is imperative for the development of effective prevention programs. Among the most consistent and important factors related to adolescent drinking are social influences [5–7]. Peer relationships constitute a social context in the development of young people; children and teenagers are particularly susceptible to peer influence due to the enormous importance of peers at this developmental stage [8–10].

A strong association between people’s drinking behavior and the drinking behavior of their friends has been documented in many studies [11–14]. There are two processes that may contribute to this association: social influence, in which a person changes their behavior to be more in line with the behavior of their friends, and social selection, in which people tend to befriend those who engage in similar behaviors. It is generally understood that these processes often occur simultaneously [15–17]. Therefore, the estimation of influence effects must consider selection effects, and without knowledge of the latter, the effect size of the former cannot be estimated correctly [18, 19].

The difficulty of statistically disentangling peer influence from a tendency to associate with similar others has long been recognized. Some of the methods that have been attempted to resolve this issue are cross-lagged panel models [20–22], discrete time event history models [23], and two stage least square regression [24, 25]. All of these methods, however, have inherent shortcomings, in that they do not adequately address the network dependence of the actors, they fail to consider potentially important unobserved feedback mechanisms between networks and behavior, and they do not control for alternative mechanisms that may be responsible for observed changes [26].

Two important advances in methodology have given a boost to the area of peer influence studies. The first involves behavior measurement methods. Traditionally, peer behavior has been measured subjectively—that is, respondents are asked for their evaluations of their friends’ behavior. The drawback of this approach is that it does not measure peer behavior so much as the respondent’s perception of it. Therefore, this measure is prone to projection bias, which describes situations in which respondents project their own behavior onto that of their friends. Bauman and Ennett in their review [15] demonstrate that in the studies where both measures (perceived reports of friend’s drinking and smoking and actual reports) were used, perceived reports were much more strongly correlated with respondent’s own behavior than actual reports. Studies that use surveys with network (sociometric) components can offset this problem. With this method, respondents report on their own behavior, and sociometric component shows who is friend with whom. This design can provide objective, rather than subjective, information about the behavior of the respondents as well as that of their immediate peers [15, 16].

The second advance is a methodological innovation in modeling influence and selection that allows an accurate disentanglement of these intertwined processes. A new class of models, called stochastic actor-oriented models (SAOM), has been developed and improved upon since its introduction by Tom Snijders in 2001 [27–29]. A main distinction of these models, compared to previous approaches, is that they simultaneously and explicitly model the co-evolution of social networks and actors’ behavior [26]. Data is collected from a complete network, meaning that every actor within a designated boundary (i.e., school class or grade) is surveyed. Estimations from such models require at least two waves of panel network data; at each wave, actors’ behavior and the ties between actors are measured. Actors create, maintain, or disrupt ties; they may also change their behavior. The unobserved process of change takes place in micro-steps, with the alteration of one tie at a time or the movement of one step at a time on a behavioral scale. There are separate parts of model specification: one for network change, another for behavioral change. This allows separate conclusions to be drawn regarding selection and influence processes that occur in networks. The development of this approach, together with the availability of free software (first stand-alone SIENA, then R package RSIENA) [29], has stimulated research of social influence in diverse fields. Over the last 20 years empirical researchers have applied SAOMs in many areas of research, including peer influence studies, for which they are particularly suited [30, 31]. Dozens of studies using SAOM methods to examine adolescent drinking behavior have now been published, which makes it possible to aggregate their results in meta-analysis.

We conducted a systematic review and meta-analysis of SAOM studies of adolescent drinking behavior. The goals of this study are: 1) to measure the average effects of peer influence and peer selection on the drinking behavior of adolescents, and 2) to understand which covariates (e.g., age, parental control) and study characteristics (e.g., network size, interval between waves) affect the magnitudes of these effects.

## Methods

### Study eligibility criteria

This review was conducted following PRISMA guidelines [32] (**S1 Appendix**) and carried out following a published protocol [33] (PROSPERO registration number CRD42019119836). All English language studies published between January 1st, 2001 and May 15th, 2020 were included. The time frame was determined by the fact that the first article that described SAOM methodology was published in 2001 [27]. The criteria included studies with a longitudinal design (at least two waves), studies that use SAOM methodology for analysis, studies that have a sociometric component (i.e., explicit measuring of social ties/friendship between participants), studies in which the behavior modeled in SAOM is alcohol consumption, and studies in which the study population is children or adolescents (with the age of participants between 11–19 years old).

### Information sources and search strategy

Studies published in academic journals, dissertations, reports, and conference materials were gathered through searches of the following electronic databases: Web of Science, Scopus, PubMed, the Cochrane Library—including the Cochrane Database of Systematic Reviews and the Cochrane Central Register of Controlled Trials (CENTRAL), EBSCOhost (including MEDLINE, SocINDEX, Academic Source, and ERIC), PsycINFO (PsycNET), the Excerpta Medica database (Embase), and the Cumulative Index to Nursing and Allied Health Literature (CINAHL). To minimize the possibility of publication bias, a search of theses and dissertations was included (ProQuest Dissertations and Theses Global). A Google Scholar search was used to check for unpublished studies and gray literature, and conference abstracts of thematically relevant conferences (such as Sunbelt 2001–2019 and EUSN 2016–2019) were hand searched; authors were also contacted to identify any unpublished studies.

The search queries included an extensive list of synonyms corresponding to the following three points of interest: SAOMs methodology, drinking behavior, and adolescents. Point 1, analytical methodology, included terms like "longitudinal network analysis", "stochastic actor-oriented models", "stochastic actor-based models", "SAB model", "SABM", "SAOM", "RSIENA" and other relevant terms (see S2 Appendix for complete list of synonyms). Point 2, drinking behavior, included “alco”, “binge”, “drink”, “substance abuse” and other synonyms for alcohol use. Point 3 included words “adolesc*”, “youth”, "young", “student” and other synonyms for young people.

The search queries were formulated for the Web of Science database and adapted for other databases. Full search query is available in **S2 Appendix**.

### Risk of bias

To minimize publication bias, we included unpublished studies—dissertations and conference theses—in our analysis. We contacted the authors of unpublished studies (with up to three email attempts) and discovered that most of them had indeed been published. We tested for publication bias using modification of Egger’s regression test for dependent effect sizes [34].

In the absence of an approved checklist for longitudinal panel network studies, we used several markers to assess the risk of bias for individual studies: the number of networks, the number of participants, the amount of time between waves, the response and attrition rate, the percentage of missing data. Because the RSIENA package includes an imputation procedure that replaces missing data using information from the previous or next wave or the global average, studies with up to 10% of missing data is not a problem for SAOM models [29].

### Study selection

Lists of articles were retrieved from the electronic databases and checked for duplicates. The articles were classified as “relevant,” “irrelevant,” or “unclear,” based on titles and abstracts. Articles considered irrelevant if they did not have a longitudinal design; or did not explicitly measure social ties between participants; or did not use stochastic actor-oriented models for analysis; or did not analyze alcohol consumption; or the study population was not children/ adolescents. Simulation studies without real data, theoretical and review articles also considered irrelevant for meta-analysis. Articles classified as “irrelevant” were removed. The full texts of the remaining articles were carefully examined to determine whether they should be included in the meta-analysis.

**Fig 1:**
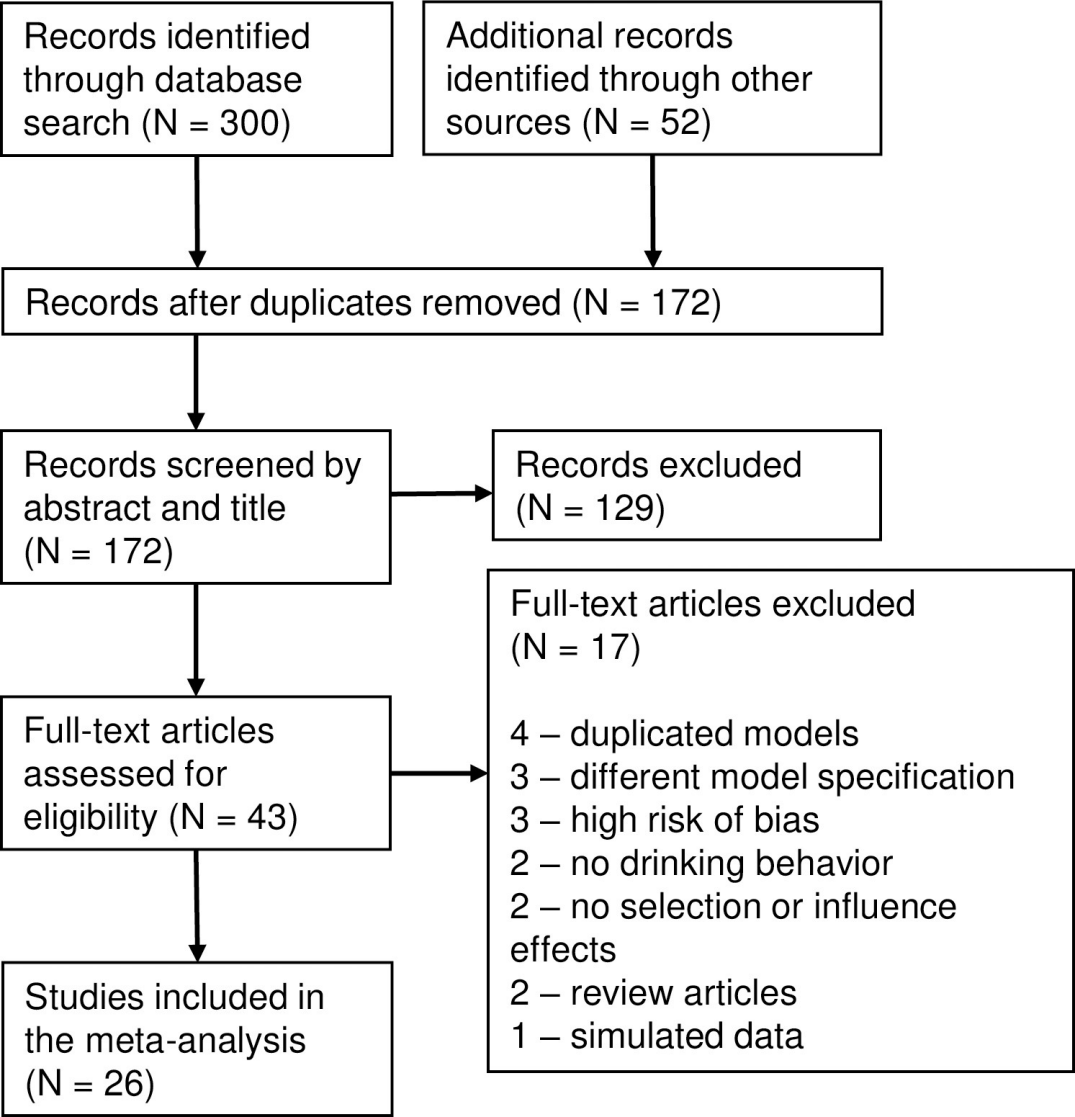
Flowchart diagram for articles screening and selection.

The flowchart diagram (Fig 1) displays the step-by-step process of article selection. Searches of the electronic databases allowed for the collection of 300 articles. Additional searches of Google Scholar and Sunbelt/EUSN conference abstracts added another 52 studies. After duplicates were removed, 172 articles were screened based on abstract and title, and of these, 129 were excluded; 17 more were excluded after full-text screening. Four studies were excluded because they presented duplicated models: first as dissertations, then as published articles. Other reasons for exclusion were different type of risk behavior (e.g., summing up scales for drinking, smoking, and marijuana use) or high risk of bias (too small sample size, too high percentage of missing data). One article published in 2006 [35] did not include quadratic shape effect which made its results unreliable (Tom Snijders, personal communication). In the dissertation of K. Coronges [36] about 40% of network data were missing; in the paper of Knecht et al. [37] the models did not converge; and in the dissertation of S. Peterson [38] the interval between waves was just 2 weeks that made it incomparable with all other studies. 26 studies we included in the meta-analysis [39–63].

### Data collection

The following items were extracted from the full texts of the selected articles:

1. Bibliometrics: authors, title, source, year of publication.
2. Characteristics of the study: country(s) and year in which the study was conducted, project title/database title, sample size (number of participants and number of networks), type of sample (general sample or at-risk group), age of participants.
3. Study design: number and frequency of survey waves (and lag between waves), data collection techniques, name generator questions (types of social ties and maximum number of nominations).
4. Description of alcohol use behavior (frequency of drinking, binge drinking etc.) and the number of categories in the alcohol measuring scale.
5. Covariates included in the model: gender; age; personality characteristics; other behaviors (smoking, physical activity etc.); family and school factors (parental control, parental drinking, school level of alcohol use etc.)
6. Response rate and attrition rate, description of missing data, treatment of missing data.
7. Model specification: influence and selection effects; structural effects.
8. Effect size data: parameter coefficients (log odds ratios) and standard errors for selection and influence effects, behavior dynamics effects, network structural effects.

Characteristics of the selected studies are presented in **S3 Appendix**.

### Data synthesis

The SAOM models estimated in RSIENA can include different specifications of influence and selection effects. The influence effects can be specified by "average similarity" (avSim), "total similarity" (totSim), or "average alter" (avAlt) [29]. Since they are calculated and interpreted in distinct ways, we conducted three different meta-analyses, separately for each specification of influence effect.

The selection effects can be specified either by similarity/identity effects (simX/sameX), or by the covariate-ego × alter effect (egoXaltX) that have to be analyzed separately. We conducted meta-analysis only for simX specification because the egoXaltX specification has been used very rarely (only in 4 articles).

Our data had 3-level nested structure because some articles provided more than one model (separate models for different schools or age cohorts), and several papers were based on the same project/database. To account for non-independence among effect sizes we used multilevel random-effects (MLRE) models with random effects at the coefficient level, the article level, and the database level. For model estimation we employed the metafor package in R (rma.mv) followed by estimating cluster-robust standard errors and confidence intervals [64].

Coefficients of SAOM models are presented as log odds ratios; hence, the mean effect sizes were calculated as logs odds ratios. To provide an effect size evaluation in metrics most often used in meta-analysis [65], we converted average log odds ratios into Cohen’s d.

To account for heterogeneity in effect sizes we pre-specified several moderators: number of networks, network size, number of possible nominations, between-waves time, country, gender composition and socio-economic status of the sample. We followed the practical recommendation to use at least ten studies for each covariate in the meta-regression [65].

Supplementary analyses were conducted on subsets of models (with one article per project/database).

Datasets, codes, and supplementary analyses results are available at https://osf.io/3avsn.

### Patient and public involvement

This research was based on analyses of previously published studies and did not involve direct patient and/or public involvement.

## Results

Twenty-six articles were selected for meta-analysis. All the studies were conducted in middle or high schools, and all the studies used similar data collection techniques: survey questionnaire with a sociometric part (friendship nomination). Fifteen articles had been based on the USA samples. The Add Health (USA) from 1994 was the earliest study, and two studies from 2012 (the Netherlands and Russia) were the most recent ones. Eight articles analyzed data from the Add Health project, and five articles—from the PROSPER project. The articles based on the same dataset used different subsamples, different specifications of the influence and selection effects, or included different additional covariates in the model (see **S3 Appendix**). Several articles reported more than one model: either models for different subsamples (schools, age cohorts) or models with different number of covariates. We collected coefficients from all the models for different subsamples, but we took only one model from the models with different number of covariates. It is important to note that for modeling network and behavior change all articles used evaluation function; thus, the models were comparable.

### Peer influence

**Fig 2:**
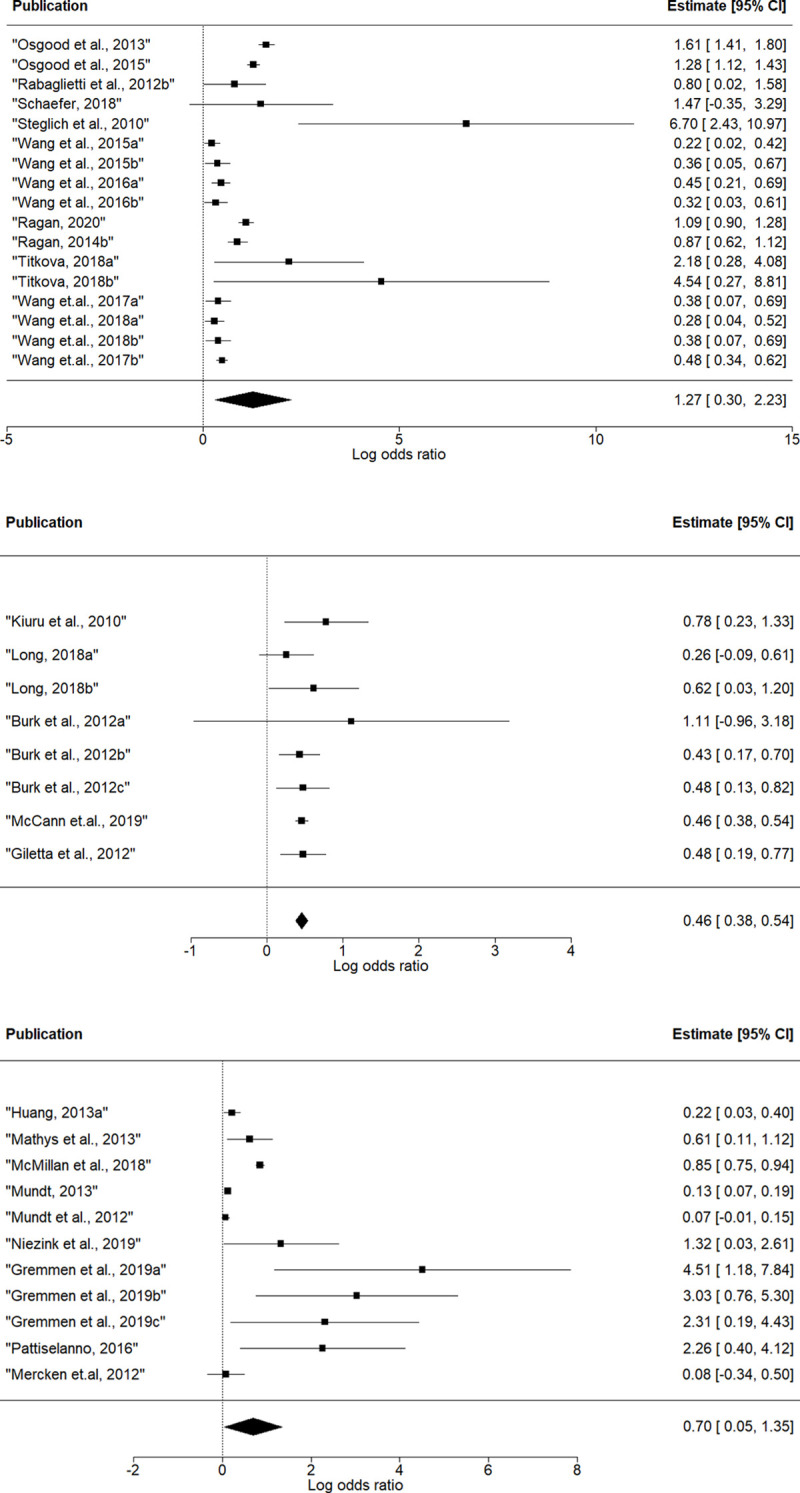
Forest plots of effect size estimates for peer influence effects: (a) avSim, (b) totSim, (c) avAlt.

Meta-analyses for different specifications of influence effect were conducted separately. Results are presented in Fig 2 and Table 1.

**Table 1:** Effect size estimates for peer influence effects.

	Log odds ratio (95%CI)	Cohen’s d	N models	N articles	N databases	Q
**avSim**	1.27* (0.04; 2.49)	0.70	17	12	5	236.7***
**totSim**	0.46*** (0.44; 0.48)	0.25	8	5	5	3.3
**avAlt**	0.70’ (-0.01; 1.41)	0.39	11	9	7	222.1***

Note: **‘**p<0.1;

*p < 0.05; **p < 0.01;

***p<0.001.

avSim–average similarity effect.

totSim–total similarity effect.

avAlt–average alter effect.

Average similarity effect was used in 17 models nested in 12 articles and 5 databases; total similarity effect was used in 8 models nested in 5 articles and 5 databases; and average alter effect was used in 11 models nested in 9 articles and 7 databases. The mean effect size was positive for every specification of influence effect, albeit with different significance level. For total similarity, the overall effect was highly significant (mean log odds ratio = 0.46, p < 0.001, 95% CI = [0.44; 0.48]), and Q statistic demonstrated that there was no heterogeneity between individual model coefficients. For average similarity, the mean effect size was also significant (mean log odds ratio = 1.27, p < 0.01, 95% CI = [0.04; 2.49]), with significant heterogeneity test (Q = 236.7, p < 0.001). For average alter the mean effect was significant on 10% level (mean log odds ratio = 0.70, p = 0.053, 95% CI = [-0.01;1.41]), with significant heterogeneity test (Q = 222.1, p < 0.001). Convergence log odds ratio into Cohen’s d gives 0.70 for average similarity, 0.25 for total similarity, and 0.39 for average alter effect. The results signify that adolescents adjust their drinking behavior to match the behavior of their friends.

In the reviewed articles, adolescent drinking behavior was measured using 3-, 4-, 5-, 6-, or 7-point scales. For the interpretation of our results in terms of one unit of behavior change, we rescaled the coefficients for average similarity and total similarity by dividing individual log odds ratios and their standard errors by (k-1), where k is the number of points in the scale. New models were estimated using the converted effect sizes. Rescaled coefficients were as follows: for avSim: log odds ratio 0.56, odds ratio 1.76, p = 0.09; for totSim: log odds ratio 0.12, odds ratio 1.13, p = 0.003. For average similarity effect odds ratio value 1.76 means that the chances that a teenager will move one unit closer to the mean level of alcoholic behavior of their friends is 76% higher than chances they will not change their behavior. For total similarity effect, odds ratio value 1.13 means that having one additional friend who drinks more than a teenager raises the probability of an increase in drinking as compared to no change by 13% (for details, see Ripley, Snijders et al., 2020, p. 183–185 [29]). Interpretation of average alter effect is different: the estimated parameter 0.70 means that when comparing a person whose friends are 0.70 units higher on the drinking scale than friends of another person, the odds of increasing drinking behavior compared to no change are two times higher (exp(0.70) = 2.0) for the first individual than for the second one.

Testing for moderation effects was not possible because of limited number of studies for each influence effect specification. Moderation analysis is not recommended when the number of studies is small. Common recommendation is at least ten studies for each moderator, and even ten may be too few when the covariates are unevenly distributed across studies [65, 66].

### Peer selection effect size

**Fig 3:**
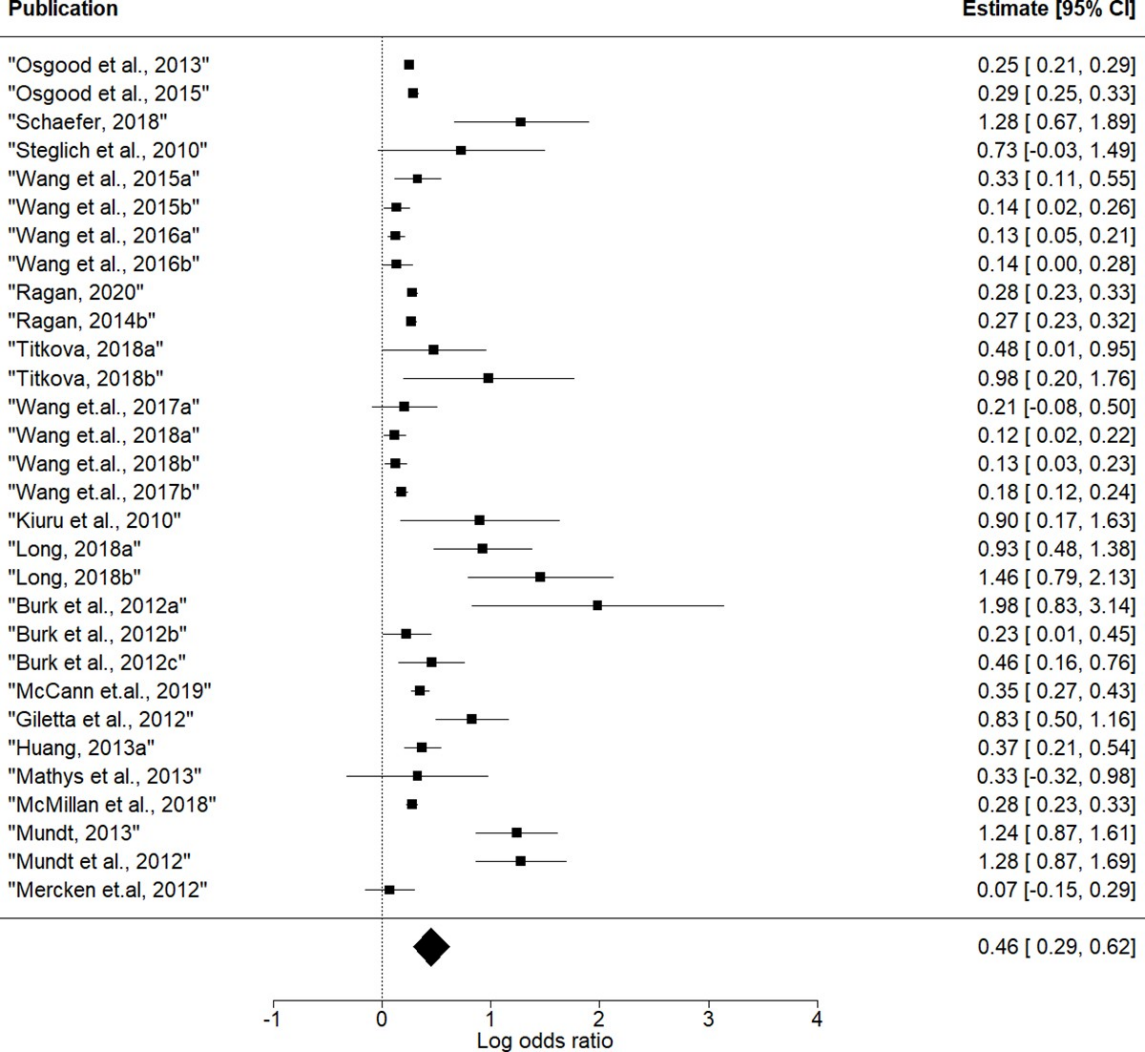
Forest plot of effect size estimates for peer selection.

Thirty effects of peer selection (simX) were nested within 22 articles and 11 databases. Drinking similarity could be included in the model with any of three specifications of influence effect. To account for this, we used the influence effect specification as a moderator in meta-regression. The results are presented in Fig 3 and Table 2.

**Table 2:** Effect size estimates for peer selection effect (simX).

	Log odds ratio (95%CI)	Cohen’s d	Q	F
**Mean Effect Size**	0.46*** (0.28; 0.63)	0.25	164.4***	
**Influence**			141.0***	1.9
**Intercept (ref. avAlt)**	0.55‘ (-0.06; 1.15)			
**avSim**	- 0.28 (-1.00; 0.44)			
**totSim**	009 (-0.44; 0.62)			

Note: **‘**p<0.1; *p < 0.05; **p < 0.01;

***p<0.001

avSim–average similarity effect.

totSim–total similarity effect.

avAlt–average alter effect.

The overall selection effect was positive and significant: log odds ratio = 0.46, p < 0.001, 95% CI = [0.28; 0.63]. Recalculating the overall effect from the log odds ratio to Cohen’s d gives d = 0.31. The result means that adolescents prefer to select friends who are similar to them with in terms of alcohol consumption. ту.

Rescaling coefficients for the interpretation in terms of one-unit changes in behavior gives a log odds ratio of 0.12, p < 0.001, and odds ratio = 1.13. The interpretation of this result is that a teenager has 13% higher odds of befriending someone who has the same score on the drinking behavior scale than someone who is one unit away on this scale.

The Q statistic reflected large heterogeneity in selection effect sizes (Q = 164.4; p < 0.001). The heterogeneity was partly explained by the influence effect specification, but still remained large (residual heterogeneity Q = 141.0, p < 0.001). F statistics indicates that influence effect specification does not moderate the effect of peer selection. In other words, there is no difference in selection effect coefficient in models with different specifications of peer influence effect.

Other moderators could not be tested due to small number of studies for each potential moderator.

### Publication bias and sensitivity analyses

Egger’s regression tests for dependent effect sizes and funnel plots were used for publication bias detection. While no bias was detected, it should be noted that such tests do not have strong power, especially when the number of articles is small, as in case of our meta-analysis [34].

Robustness checks for outliers and influential cases were performed for each specification (avAlt, avSim, totSim, simX); no outliers and/or influential cases were detected [64, 65].

Many studies in out meta-analysis used the same databases: for avAlt effect, two papers were based on the SNARE data and two on the Add Health data; for avSim effect, four papers were based on the PROSPER data and five on the Add Health data; for simX similarity effect, eight articles were based on the Add Health data and five articles on the PROSPER data. To eliminate dependencies between effect sizes originated from the same database, we estimated models where only one article for each database was selected. The sensitivity analyses results have shown that all the coefficients were withing the limits of 95% confidence intervals of the main analyses reported in Tables 1 and 2. Specifically, avSim coefficient was 1.29 (95% CI = [0.07; 2.50]); avAlt coefficient was 0.47 (95% CI = [0.05; 0.88]), and simX coefficient was 0.52 (95% CI = [0.30; 0.74]).

Detailed results of publication bias analyses and sensitivity analyses are available at https://osf.io/3avsn.

## Discussion

This article is the first meta-analysis of adolescent drinking behavior studies that employ a longitudinal network design and SAOM for a rigorous statistical unraveling of the co-occurring processes of peer selection and peer influence. The results demonstrate that both the selection effect and influence effect are significant, which indicates that adolescents prefer to select friends who are similar to them with regard to alcohol consumption, and that adolescents adjust their drinking behavior to match their friends’ behavior.

It is interesting to compare our results to those reported in a recent meta-analysis of SAOM studies of a different behavior [67]. Owen Gallupe, John McLevey, and Sarah Brown (2019) [67] analyzed studies of illegal offending/delinquent behavior in adolescents. They meta-analysed only articles with most often specification of the influence effect (avSim). The mean effect size from this research is very similar to ours: log odds ratio = 1.23 (Cohen’s d = 0.68). Such consistency in results of two independent meta-analyses is quite remarkable, considering that developmental psychologists predicted that the strength of peer influence may substantially differ for different behaviors [68, 69].

### Strengths, limitations, and future directions

The strength of the current study is the high reliability of its meta-analysis results. Several sensitivity analyses demonstrated the robustness of our findings. Publication bias is unlikely, as was confirmed through analysis. All of the studies selected for our meta-analyses used state-of-art methodology, which allows rigorous statistical disentangling of the two simultaneously occurring processes of peer influence and peer selection. There is a certainty that the estimation of average effect sizes for influence and selection is robust.

The main limitation of our study is the small number of individual studies that were included in the meta-analysis. While the estimation of the mean effect size is robust, there was not enough data to analyze the role of important covariates: individual and family characteristics, and also important network characteristics, in particular network size and density. Developmental researchers point out that age differences in susceptibility to peer influence are substantial; the principal conclusion is that resistance to peer influence increases during adolescence [70, 71], and findings on gender differences in susceptibility to peer influence are inconsistent [72]. More longitudinal network studies that collect primary data will be necessary to further investigate the role of these important characteristics.

The second limitation of this research is that most of the studies were conducted in the USA and some European countries, which limits its extent of generalization. More research in different countries would allow us to generalize these findings to youth from different cultural backgrounds. The third limitation relates to the fact that most databases used in the reviewed studies were relatively old; for example, the Add Health study began in the early 1990s. It is not unreasonable to expect that almost 30 years later, the drinking behavior of teenagers would have changed. The fourth limitation stems from the nature of the networks investigated by researchers. In all the studies, peer influence was restricted to the influence of school friends, while it is obvious that the social networks of adolescents are more complex and often include friends outside of school. Moreover, in modern times, the ubiquity of online social networks makes the structure of adolescent social networks, and arguably, social influence itself, even more complex.

Our review has several implications for future research. It is worth noting that none of the reviewed studies explicitly measured susceptibility to peer influence, despite the fact that developmental psychologists detected considerable individual variability for this characteristic [73–75]. Presently, these two areas of peer influence research are completely separate: psychologists study susceptibility/resistance to peer influence using self-report measures (e.g., the Resistance to Peer Influence questionnaire) [70], but there is no information on how different levels of self-reported susceptibility/resistance relate to changes in an individual’s behavior under peer pressure. Network researchers, on the other hand, measure behavioral changes due to peer influence, but there is no complementary information on differences in susceptibility/resistance. We suggest that bringing these two approaches together will enrich both sides.

Another implication involves health-promotion interventions. Schools are perfect settings for prevention programs, but such programs are often less effective than intended [76]. There is a growing recognition of the benefit of using social networks in behavioral intervention [77–79], and it is likely that interventions that are based on a detailed understanding of the complex social system of adolescents would be more effective. In school-based settings, peer influence may increase the efficiency of prevention and intervention programs. Further research into social networks with a focus on peer influence should inform practitioners and policy makers in their design and delivery of effective prevention programs.

## Supporting information

S1 Appendix Prisma checklist. (DOCX)

S2 Appendix Search query. (DOCX)

S3 Appendix Characteristics of the selected studies. (XLSX)
