# Supplementary material for: Peer influence in adolescent drinking behavior: A meta-analysis of stochastic actor-based modeling studies
Source: PLoS One. 2021 Apr 16;16(4):e0250169. doi: 10.1371/journal.pone.0250169 (PMC8051820; doi:10.1371/journal.pone.0250169)
Supplement: S2 Appendix — (DOCX) [file pone.0250169.s002.docx]

**S2 Appendix.**

**For Web of Science and Scopus**

( ( alcopop* OR alcohol* OR liquor OR liqueur OR wine OR beer OR binge OR drunk OR drink* OR boost OR booze OR tipple OR spirits OR beverage OR cocktail* OR "substance us*" ) AND ( adolesc* OR "young adult*" OR youth OR "young person*" OR "young people" OR teen* OR student* OR "peer group*" OR child* OR pupil* OR junior ) AND ( ( "Rsiena" OR "R-Siena" OR "longitudinal network anal*" OR "simulation investigation for empirical network*" OR "stochastic actor-oriented" OR "actor-oriented model*" OR "actor-oriented analys*" OR "stochastic actor-based" OR "actor-based model*" OR "actor-based analys*" OR "stochastic network model*" OR "stochastic network analys*" OR "SAOM" OR "SAOMs" OR "SAO model*" OR "SAB model*" OR "SABM") OR ( network AND select* AND influen* AND ( dynamic* OR longit* OR co*evolution ) ) OR ( ( siena AND network ) AND NOT ( italy OR city ) ) ) )

**for ProQuest**

NOFT((alcopop* OR alcohol* OR liquor OR liqueur OR wine OR beer OR binge OR drunk OR drink* OR boost OR booze OR tipple OR spirits OR beverage OR cocktail* OR "substance use" OR "substance using") AND (adolesc* OR "young adult" OR youth OR ("young person" OR "young persons") OR "young people" OR teen* OR student* OR ("peer group" OR "peer groups") OR child* OR pupil* OR junior) AND (

("Rsiena" OR "R-Siena" OR "longitudinal network anal*" OR "simulation investigation for empirical network" OR "stochastic actor-oriented" OR "actor-oriented model" OR "actor-oriented anal*" OR "stochastic actor-based" OR "actor-based model*" OR "actor-based anal*" OR "stochastic network model*" OR "stochastic network anal*" OR "SAOM*" OR "SAOMs" OR "SAO model*" OR "SAB model*" OR "SABM") OR (network* AND select* AND influen* AND (dynamic* OR longit* OR co*evolution)) OR (((siena OR "SIENA") AND network*) NOT (italy OR city))))

**For　Cochrane　library**

( ( alcopop* OR alcohol* OR liquor OR liqueur OR wine OR beer OR binge OR drunk OR drink* OR boost OR booze OR tipple OR spirits OR beverage OR cocktail* OR "substance us*" ) AND ( adolesc* OR "young adult*" OR youth OR "young person*" OR "young people" OR teen* OR student* OR "peer group*" OR child* OR pupil* OR junior ) AND ( ( "Rsiena" OR "R-Siena" OR "longitudinal network anal*" OR "simulation investigation for empirical network*" OR "stochastic actor-oriented" OR "actor-oriented model*" OR "actor-oriented analys*" OR "stochastic actor-based" OR "actor-based model*" OR "actor-based analys*" OR "stochastic network model*" OR "stochastic network analys*" OR "SAOM" OR "SAOMs" OR "SAO model*" OR "SAB model*" OR "SABM") OR ( network AND select* AND influen* AND ( dynamic* OR longit* OR co*evolution ) ) OR ( ( siena AND network ) NOT ( italy OR city ) ) ) ):ti,ab,kw
